# Glutathione boosting the cytotoxicity of a magnetic platinum(iv) nano-prodrug in tumor cells†
†Electronic supplementary information (ESI) available: Supplementary Fig. S1–S8, Tables S1–S4 and experimental procedures. See DOI: 10.1039/c5sc04049c


**DOI:** 10.1039/c5sc04049c

**Published:** 2016-01-20

**Authors:** Zhenzhu Zhu, Zenghui Wang, Yigang Hao, Chengcheng Zhu, Yang Jiao, Huachao Chen, Yun-Ming Wang, Jun Yan, Zijian Guo, Xiaoyong Wang

**Affiliations:** a State Key Laboratory of Coordination Chemistry , School of Chemistry and Chemical Engineering , Nanjing University , Nanjing 210023 , P. R. China . Email: zguo@nju.edu.cn ; Fax: +86 25 83314502 ; Tel: +86 25 89684549; b State Key Laboratory of Pharmaceutical Biotechnology , School of Life Sciences , State Key Laboratory of Analytical Chemistry for Life Science , Nanjing University , Nanjing 210023 , P. R. China . Email: boxwxy@nju.edu.cn; c Department of Biological Science and Technology , Institute of Molecular Medicine and Bioengineering , National Chiao Tung University , No. 75 Bo-Ai Street , Hsinchu 300 , Taiwan; d State Key Laboratory of Pharmaceutical Biotechnology , MOE Key Laboratory of Model Animals for Disease Study , Model Animal Research Center of Nanjing University , Nanjing 210061 , P. R. China

## Abstract

Superparamagnetic iron oxide nanoparticles (SPIONs) are potential vehicles for targeted drug delivery and viable contrast agents for magnetic resonance imaging (MRI). A Pt^IV^ prodrug (HSPt) derived from functionalization of cisplatin with hydroxyl and succinate is conjugated with a poly(ethylene glycol) (PEG)-modified SPION for cancer therapy and monitoring of therapeutic responses. The relaxivity of HSPt–PEG-SPIONs is larger than that of commercial contrast agent Feridex, and a tumor-selective negative contrast is observed in MRI in a magnetic field. HSPt–PEG-SPIONs can be dissociated and reduced into Pt^II^ species by glutathione (GSH). Instead of forming DNA–Pt crosslinks, the reduced product induces direct DNA single- or double-strand breaks, which is uncommon for Pt drugs. The cytotoxicity of HSPt–PEG-SPIONs is positively correlated with the GSH level of tumor cells, which is opposite to the scenario of current Pt drugs. HSPt–PEG-SPIONs are as cytotoxic as cisplatin against cancer cells but are almost nontoxic towards normal cells. Since the mechanism of action of the nanocomposite is different from the established paradigm for Pt drugs, it may become a special theranostic agent for cancer treatment.

## Abstract 2

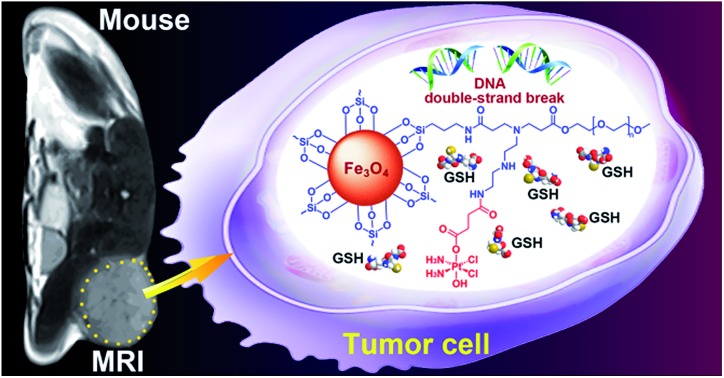
Fe_3_O_4_ nanoparticles with Pt-prodrug payloads display MRI capability and tumor-specific cytotoxicity correlating positively with glutathione-mediated DNA cleavage and reduction.

## Introduction

Cisplatin is a first-line chemotherapeutic drug against a variety of cancers.^1^ However, its application has been heavily conditioned by severe systemic toxicities like nephrotoxicity and neurotoxicity.^2^ In addition, the efficacy of cisplatin is limited because of inherent or acquired drug resistance.^3^ These defects mainly result from its indiscriminate body distribution and insufficient tumor accumulation, and also from its detoxification by sulfur-containing biomolecules.^4^ Targeted prodrug systems have been proven effective in minimizing the systemic toxicity and in maximizing the tumor accumulation of Pt drugs.^5^ SPIONs could guide drugs preferentially to the biological target through an external magnet and provide a strong negative contrast effect in *T*_2_-weighted MRI;^6^ and polymer-modified SPIONs possess some excellent properties, such as hydrophilicity, nontoxicity, and nonimmunogenicity, for drug delivery.^7^ Therefore, in the past few years we endeavoured to load Pt^II^ moieties onto SPIONs as theranostic agents for simultaneous therapy and diagnosis.^8^ However, Pt^II^ moieties are capable of reacting with bionucleophiles during the delivery, and hence could be toxic to normal tissues.

Octahedrally coordinated Pt^IV^ complexes are substantially more inert than Pt^II^ complexes and thus can avoid the undesirable side reactions in the blood plasma.^9^ Likewise, they are inactive towards nuclear DNA, so the reduction of Pt^IV^ complexes to kinetically labile Pt^II^ species is necessary to exert their cytotoxic effects. GSH is one of the intracellular reductants that activate Pt^IV^ complexes.^10^ Many studies have shown that cisplatin-based Pt^IV^ complexes can be reduced to reactive Pt^II^ species and bind DNA to form 1,2-d(GpG) DNA–Pt crosslinks akin to those formed by cisplatin.^11^ Meanwhile, the level of cellular GSH may decrease during the reduction, which is beneficial for attenuating its detoxifying effect on Pt^II^ species. For these potential merits, we decide to change the previous Pt^II^ pharmacophore to a Pt^IV^ complex.

**Fig. 1:**
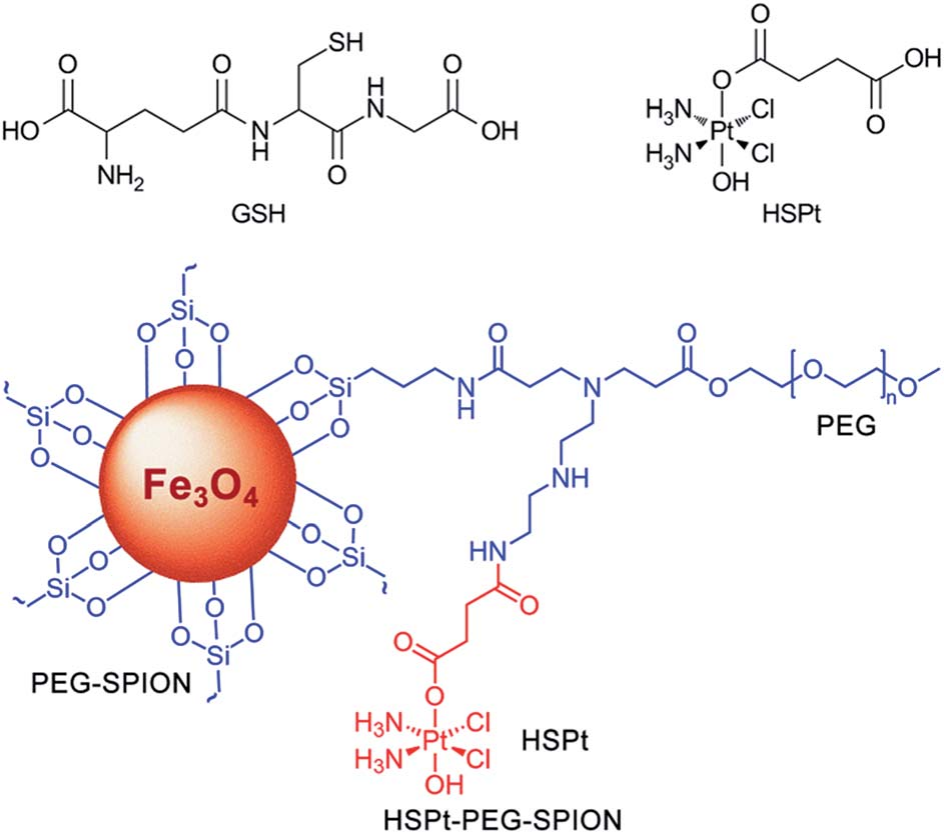
Structures of GSH, HSPt and HSPt–PEG-SPIONs.

In this study, we continue to use SPIONs (Fe_3_O_4_) to design the targeted Pt prodrug system. In order to increase the stability and stealthiness *in vivo*, SPIONs are coated with PEG, which has been proven to improve the biocompatibility, blood circulation time, and immunotherapeutic efficacy of nanoparticles.^12^ A Pt^IV^ complex, *c*,*t*,*c*-[PtCl_2_(OH)(O_2_CCH_2_CH_2_CO_2_H)(NH_3_)_2_] (HSPt), is synthesized as the pharmacophore, which is an asymmetrically functionalized prodrug of cisplatin. HSPt is loaded onto the surface of the PEGylated SPIONs, forming HSPt–PEG-SPIONs (Fig. 1). This nanocomposite exhibits some unique properties *in vitro*, such as GSH-promoted cytotoxicity against tumor cells and nontoxicity towards normal cells. Its mechanism of action also differs from that of cisplatin. Moreover, it produces a significant negative contrast in MRI, and thus could be a potential theranostic agent for chemotherapy.

## Results and discussion

### Preparation and characterization

**Fig. 2:**
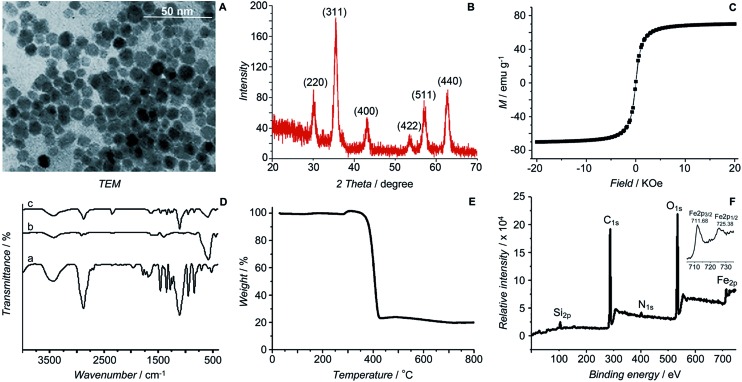
Characterization of SPIONs and PEG-SPIONs. (A) TEM image of SPIONs; (B) X-ray diffraction patterns of SPIONs; (C) field-dependent magnetization curve of SPIONs at 300 K; (D) IR spectra of (a) silane-diethyltriamine-methoxy PEG, (b) SPION/oleic acid/oleylamine, and (c) PEG-SPIONs: 3200–3700 cm^–1^ (*ν*_OH_, *ν*_NH_2__), 2885 cm^–1^ (*ν*_C–H_), 1688 cm^–1^ (*ν*_C=O_), 1539 and 1410 cm^–1^ (*ν*_COO_), 1284 cm^–1^ (*τ*_CH_2__, *ν*_Si–C_), 1201 cm^–1^ (*ρ*_OCH_3__), 1113 cm^–1^ (*ν*_Si–O–R_), 839 cm^–1^ (*ω*_NH_2__), 588 cm^–1^ (*ν*_Fe–O_); (E) TGA spectrum of PEG-SPIONs; (F) XPS spectra of PEG-SPIONs and Fe2p.

The initial SPIONs were synthesized referring to a reported method.^13^ PEG-SPIONs were obtained by modifying the SPIONs with silane-diethyltriamine-methoxy PEG, which contains an amine group in the chain. The TEM image shows that the size of the SPIONs is around 11 nm (Fig. 2A). The XRD spectrum of the SPIONs matches well with that from the JCPDS card (no. 01-1111) for the cubic-phase magnetite (Fig. 2B). The saturation magnetization (*M*_s_) of the SPIONs is 70.05 emu g^–1^ at room temperature (Fig. 2C), which is lower than that of the bulk Fe_3_O_4_ (89 emu g^–1^) but is higher than that of the modified SPIONs.^14^ The absence of coercivity and remanence indicates that the SPIONs exhibit a superparamagnetic property at room temperature. The existence of the polymer on the surface of the SPIONs is confirmed by IR spectroscopy (Fig. 2D). Thermal gravitational analysis shows that the weight of the PEG-SPIONs decreases *ca.* 80% at 300–400 °C due to the evaporation of the surface polymer (Fig. 2E). In the XPS spectrum, the PEG-SPIONs exhibit Fe2p_3/2_ and Fe2p_1/2_ photoelectron peaks at 711.68 and 725.38 eV, but no obvious charge transfer satellite is observed near the Fe2p_3/2_ peak (Fig. 2F), suggesting that Fe is basically at a mixed oxidation state of +II and +III.^15^ The ICP-MS and ninhydrin assay show that the molar ratio of free amino groups to Fe in the PEG-SPIONs is 0.146.

**Fig. 3:**
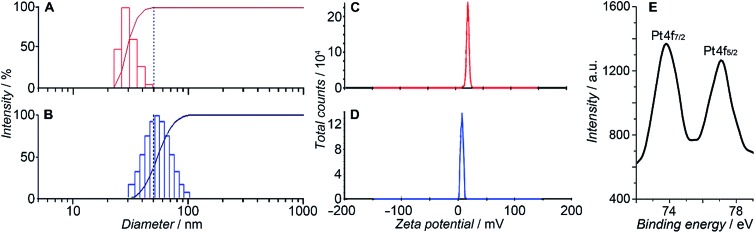
Size distribution of (A) PEG-SPIONs and (B) HSPt–PEG-SPIONs determined by dynamic light scattering; zeta potential of (C) PEG-SPIONs and (D) HSPt–PEG-SPIONs; and (E) Pt4f XPS spectrum of HSPt–PEG-SPIONs.

HSPt was derived from cisplatin by oxidation with hydrogen peroxide and substitution with succinate, successively. Owing to the existence of an amino group in the polymer and a carboxyl group in HSPt, HSPt–PEG-SPIONs were readily formed *via* EDC/NHS coupling chemistry. After conjugation, the average hydrodynamic diameter of the PEG-SPIONs increased from 31.60 ± 3.50 to 47.80 ± 5.20 nm (Fig. 3A and B), whereas the zeta potential decreased from +18.10 ± 1.30 to +1.77 ± 0.50 mV (Fig. 3C and D) due to the shielding of amine groups. These changes suggest that HSPt has bonded with the PEG-SPIONs. The weak positive potential may help the HSPt–PEG-SPIONs pass through the cell membrane (negative inside). Actually, the cellular uptake of Fe and Pt is indeed enhanced after the conjugation (see Table S1†). In the XPS spectrum of the HSPt–PEG-SPIONs (Fig. 3E), two new peaks are observed at 73.68 and 77.08 eV as compared with that of the PEG-SPIONs (Fig. 2F), which are assignable to the photoelectron peaks of Pt4f_7/2_ and Pt4f_5/2_, respectively, and are consistent with the reported binding energies for Pt^IV^ species.^16^ The maximum loading ratio of Pt to Fe (w/w) for the HSPt–PEG-SPIONs is 0.20 (see Table S2†).

### Transverse relaxivity (*r*_2_) and *in vitro* MRI

**Fig. 4:**
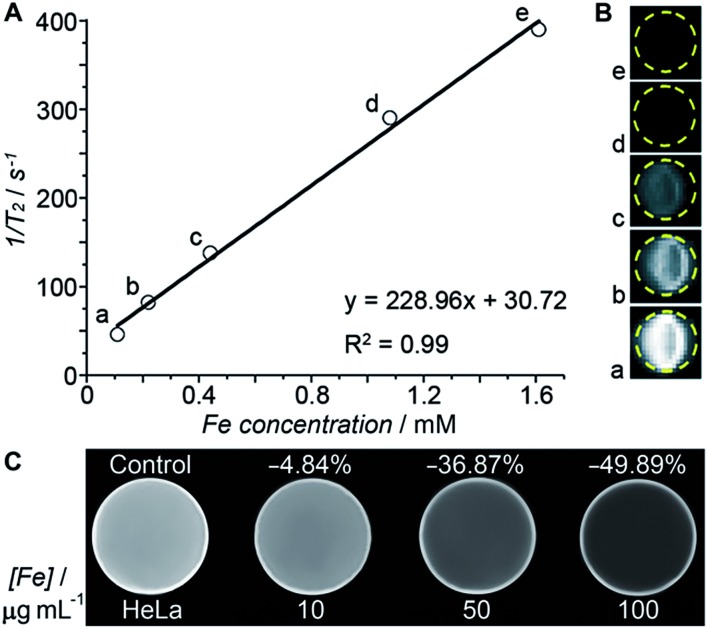
(A) The relaxation rate (1/*T*_2_) *versus* the concentration of HSPt–PEG-SPIONs (in terms of Fe), (B) *T*_2_-weighted MR images of aqueous HSPt–PEG-SPION suspensions at different Fe concentrations, and (C) *T*_2_-weighted MR images of HeLa cells after incubation with different concentrations of HSPt–PEG-SPIONs (in terms of Fe) at 37 °C for 18 h.

Transverse relaxivity (*r*_2_) represents the efficiency of a contrast agent in shortening the proton relaxation time.^17^ As Fig. 4A shows, the *T*_2_ relaxation rate (1/*T*_2_) of the HSPt–PEG-SPIONs increases linearly with the Fe concentration and the *r*_2_ value is calculated to be 228.96 mM^–1^ s^–1^, which is significantly higher than that of the *T*_2_-weighted MRI contrast agent Feridex (SPION, *r*_2_ ≈ 100 mM^–1^ s^–1^) used in clinics,^18^ and is also higher than that of similar nanocomposites.^19^Fig. 4B reveals an explicit Fe concentration-dependent darkening effect of HSPt–PEG-SPION aqueous suspensions. The *in vitro* imaging potential was examined by testing the contrast effect in HeLa cells. As Fig. 4C shows, the MR images of the cells after incubation with HSPt–PEG-SPIONs present an enhanced contrast in comparison with that of the control. The signal intensity is negatively correlated with the concentration of Fe. These results indicate that HSPt–PEG-SPIONs can effectively shorten the *T*_2_ relaxation time, enter into tumor cells and produce negative contrast in MRI.

### *In vivo* MRI

**Fig. 5:**
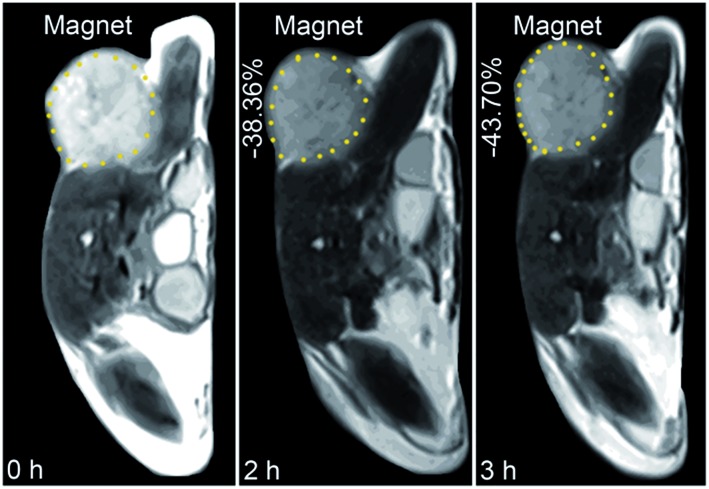
MR images of B6 mice bearing implanted RM1 murine prostate cancer before and after the injection of HSPt–PEG-SPIONs (3 mg Fe per kg) in a magnetic field.

The *in vivo* tumor-specific accumulation and imaging potential of the HSPt–PEG-SPIONs were evaluated under an external magnetic field in B6 mice bearing implanted RM1 murine prostate cancer. Fig. 5 shows the *T*_2_-weighted MR images acquired before and after the intravenous injection of the HSPt–PEG-SPIONs. A noticeable darkening effect is observed in the circled tumor area after the injection, indicating that a substantial amount of HSPt–PEG-SPIONs has accumulated within the cancer tissue. Evidently, the negative contrast enhancement is attributed to the high relaxivity (*r*_2_) of the HSPt–PEG-SPIONs. The results show that HSPt–PEG-SPIONs can target tumor tissues with the help of external magnet and achieve real-time imaging of tumor tissues during the therapy.

### Drug release

The release of Pt^II^ species from HSPt–PEG-SPIONs in the presence of GSH was monitored by ICP-MS (see Fig. S1†), HPLC and identified by ESI-MS. Peaks assignable to [Pt(NH_3_)_2_Cl(OH) + Na]^+^ and [Pt(NH_3_)_2_Cl_2_(OH)(O_2_CCH_2_CH_2_CO_2_)]^–^ are observed in the HPLC at 6 h after the reaction (see Fig. S2 and Table S3†), indicating that GSH can facilitate the dissociation of HSPt–PEG-SPIONs and reduce the Pt^IV^ prodrug into Pt^II^ species. As compared with the spectrum recorded at the start point, the intensity of HSPt and polymer peaks increased, while that of the GSH peak decreased markedly. It is known that intracellular GSH can react with cisplatin to form a Pt–GS or Pt–GS–Pt adduct, which can diminish the amount of reactive Pt^II^ species available for DNA and hence undermine the cytotoxicity of cisplatin.^20^ In this case, we only detected the [Pt(NH_3_)_2_(GS)H_2_O]^+^ adduct, which still maintains some reactivity towards DNA. The results imply that the depletion of GSH was mainly caused by the dissociation and reduction of HSPt–PEG-SPIONs, and the detoxification of Pt^II^ species by GSH is limited in this system.

**Fig. 6:**
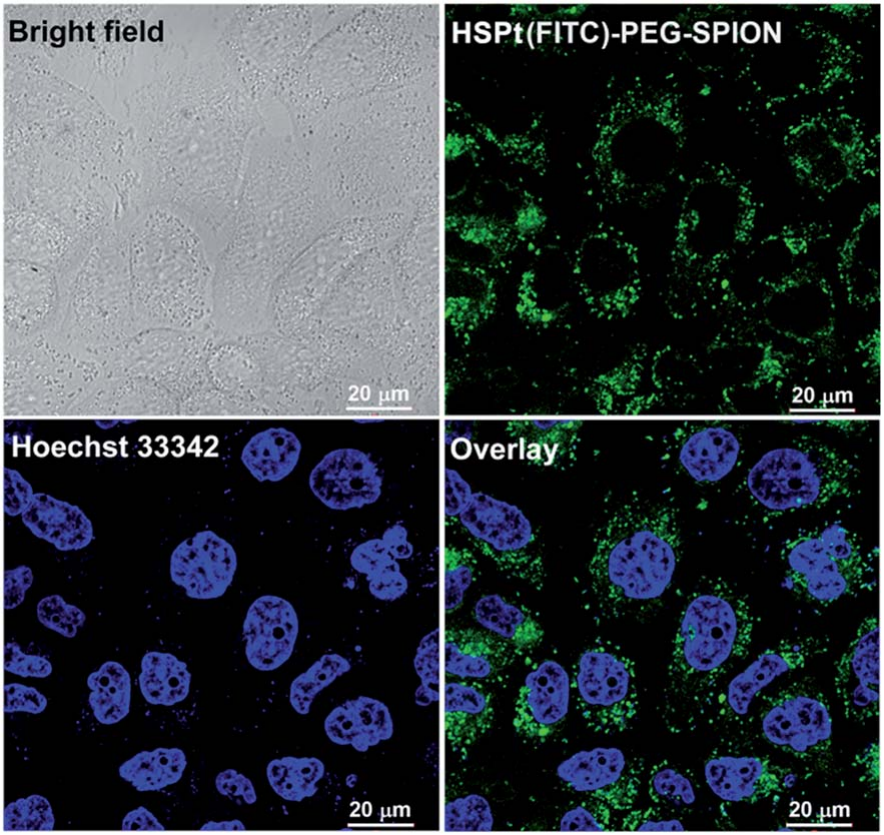
Photomicrographs of HSPt(FITC)–PEG-SPIONs (20 μM Pt) in HeLa cells observed under a confocal microscope after incubation at 37 °C for 12 h.

The dissociation of the HSPt–PEG-SPIONs in HeLa cells was monitored by confocal fluorescence microscopy. Due to the existence of surface amine groups, fluorescein isothiocyanate (FITC) could readily be conjugated onto PEG-SPIONs along with HSPt, forming the hybrid composite HSPt(FITC)–PEG-SPION; meanwhile, the fluorescence of FITC was quenched due to the Förster resonance energy transfer mechanism (see Fig. S3†).^19^ However, the fluorescence recovered as the composite was internalized by the cells (Fig. 6 *versus* S4†), showing that FITC- or HSPt-polymer moieties can become detached from the SPIONs in the presence of cellular GSH. The co-localization image after nucleolar staining with Hoechst 33342 revealed that the fluorescent FITC-polymer species or HSPt-polymer species appear only outside the nuclei, suggesting that further dissociation is required if the Pt unit is aimed at nuclear DNA.

### Interaction with DNA

**Fig. 7:**
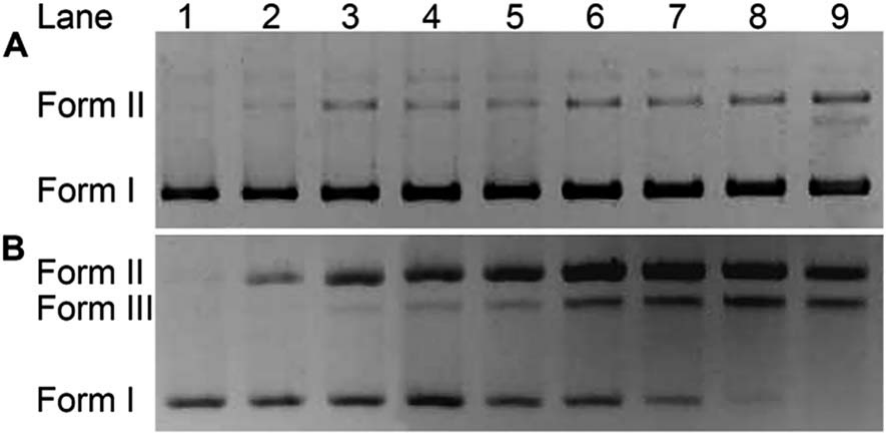
Agarose gel electrophoretic patterns of supercoiled pUC19 plasmid DNA (20 ng μL^–1^) in the presence of HSPt–PEG-SPIONs (A) without or (B) with GSH (2 mM) after incubation in buffer (50 mM Tris–HCl, 50 mM NaCl, pH 7.4) at 37 °C for 16 h. Lane 1, DNA control; lanes 2–9, DNA + HSPt–PEG-SPIONs (6, 12, 24, 30, 36, 42, 48, 54 μM, respectively).

The impact of HSPt–PEG-SPIONs on DNA was studied by agarose gel electrophoresis. As Fig. 7 shows, without GSH, HSPt–PEG-SPIONs only slightly damage the supercoiled DNA (form I), producing a finite amount of nicked DNA (form II); while cisplatin retards the migration rate of form I due to the unwinding of the DNA duplex induced by Pt binding.^21^ With GSH, HSPt–PEG-SPIONs effectively cleave form I into form II and further into linear DNA (form III); and the cleavage efficiency is positively related to the concentration of GSH (see Fig. S5†). The appearance of linear DNA demonstrates that some double-strand breaks (DSBs) have occurred. By contrast, cisplatin and HSPt only cause mild damage to DNA in the presence of GSH (see Fig. S6†). The results again suggest that the reduction product is not cisplatin, which is in accordance with the above HPLC results. It is believed that the cytotoxicity of cisplatin primarily originates from its covalent binding to DNA and subsequent inhibiting of DNA transcription.^9^ Since the reduced Pt^II^ species here do not induce DNA inter- and intra-strand crosslinks as cisplatin does, we suppose the DNA strand breaks, especially the DSBs, play a key role in the bioactivity of HSPt–PEG-SPIONs. In fact, the formation of DSBs represents the most lethal form of DNA damage, because unrepaired or misrepaired DSBs can lead to cell death and genomic instability.^22^ In current chemotherapeutic agents, only bleomycin induces direct DSBs, whereas other agents such as cisplatin induce DSBs by indirect routes.^23^ Direct DNA DSBs induced by Pt anticancer drugs were never reported before. We presumed that some reactive oxygen species (ROS) might be responsible for the cleavage and hence conducted a verification using common radical scavengers such as DMSO for ˙OH, KI for H_2_O_2_, and NaN_3_ for ^1^O_2_ (see Fig. S7†). Contrary to our assumption, the cleavage activity of the HSPt–PEG-SPIONs did not reduce markedly, suggesting that ROS may not be a determinant in the process. Thus the cleavage mechanism is not yet clear.

### Cytotoxicity

**Fig. 8:**
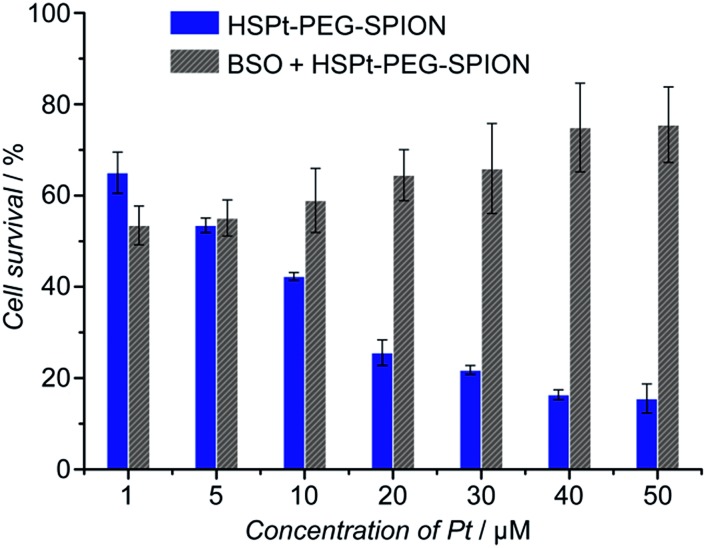
The concentration-dependent cytotoxicity of HSPt–PEG-SPIONs against the A549 cell line at 48 h before and after the pre-treatment with BSO (200 μM) for 24 h.

The cytotoxicity of the HSPt–PEG-SPIONs against human non-small lung cancer A549, human cervical cancer HeLa, and human gastric cancer SGC-7901 cell lines was tested by MTT assays. Meanwhile, the concentration of GSH in these cell lines before and after the drug treatment was measured by a GSH assay kit. The inhibition efficiency of the HSPt–PEG-SPIONs is comparable to that of cisplatin towards A549 and HeLa cell lines, but is inferior to that of cisplatin towards the SGC-7901 cell line (see Fig. S8 and Table S4†). The half-maximal inhibitory concentrations (IC_50_) of the HSPt–PEG-SPIONs and the cellular GSH concentrations are listed in Table 1. The results indicate that the IC_50_ values decrease with the concentration of GSH, that is, intracellular GSH can boost the cytotoxicity. In order to further confirm the promotive effect of GSH, the A549 cell line was pre-treated with buthionine sulfoximine (BSO), which is an inhibitor of intracellular GSH synthesis.^24^ After the treatment, the cytotoxicity of the HSPt–PEG-SPIONs was retested against this cell line. As Fig. 8 shows, the cytotoxicity was dramatically reduced because of the decrease in GSH, thus verifying the promotive effect of GSH on the cytotoxicity of the HSPt–PEG-SPIONs.

**Table 1:** IC_50_ values (μM) of HSPt–PEG-SPIONs at 48 h and intracellular concentrations of GSH (nmol per mg protein) before and after the treatment with HSPt–PEG-SPIONs

Cells	IC_50_	GSH before treatment	GSH after treatment	GSH depletion
A549	6.55 ± 3.84	48.85 ± 4.29	25.06 ± 1.44	48.7%
HeLa	10.34 ± 4.56	40.73 ± 2.56	25.47 ± 2.67	37.5%
SGC	37.52 ± 2.43	22.08 ± 2.79	16.35 ± 2.37	26.0%

It is known that GSH negatively influences the therapeutic efficacy of Pt^II^ drugs through detoxification due to its chelation with Pt^II^ centers.^20^ For Pt^IV^ prodrugs, GSH usually shows a biphasic action involving reduction of Pt^IV^ and sequent chelation with Pt^II^. However, as we demonstrated above, chelation of Pt^II^ was not observed except a limited coordination with the reduced Pt^II^ species. Therefore, the cytotoxicity of the HSPt–PEG-SPIONs is positively related to the concentration of intracellular GSH or to the depletion of GSH, as high levels of GSH are beneficial to the reduction of HSPt and the cleavage of DNA. These results imply that HSPt–PEG-SPIONs may have an advantage for overcoming the tumor resistance to cisplatin induced by GSH. It should be clarified that the depletion of cellular GSH not only resulted from the reduction of the Pt^IV^ prodrug, but also resulted from the consumption for scavenging the ROS generated through DNA damage^25^ and for forming the Pt–GS complex with Pt^II^ species. Therefore, the percentage of GSH depletion in Table 1 appears to be quite significant.

**Fig. 9:**
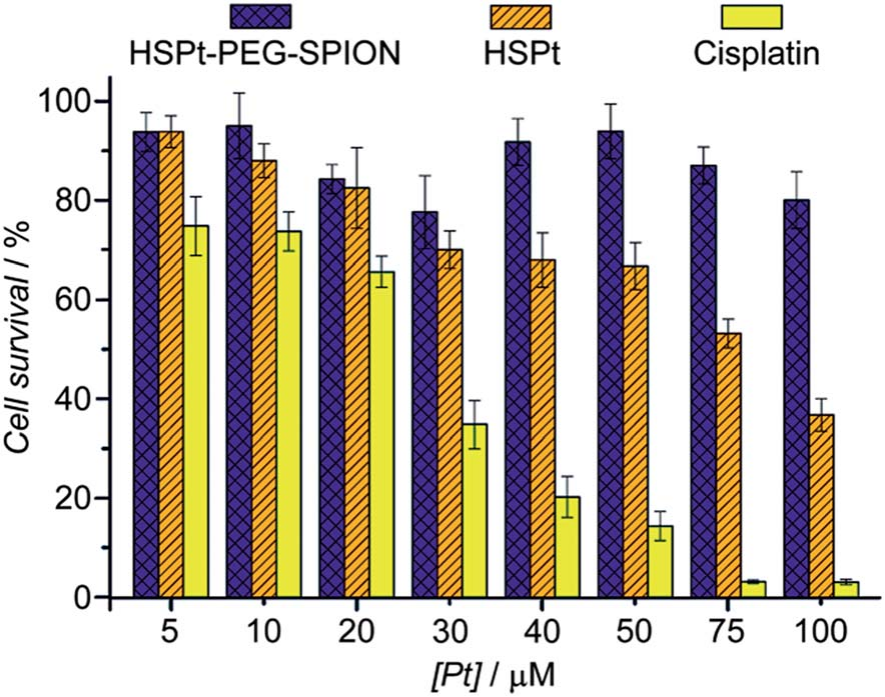
Viability of HL-7702 normal liver cells after incubation with HSPt–PEG-SPIONs, HSPt and cisplatin, respectively, at 37 °C for 48 h. Data are expressed as mean (%) ± standard deviation (S.D.) of at least three independent assays.

Finally, the biocompatibility of the HSPt–PEG-SPIONs was examined on HL-7702 normal liver cells by an MTT assay. More than 80% of the cells are alive even when the concentration reaches 100 μM, while only fractional cells are alive at this concentration for cisplatin (Fig. 9). Therefore, HSPt–PEG-SPIONs are almost nontoxic to normal liver cells.

## Conclusions

We have prepared a SPION-based nanocomposite loaded with a Pt^IV^ prodrug as a potential targeted theranostic agent for cancer treatment. In addition to magnetic-targeting and antiproliferative properties, the composite also showed low toxicity to normal cells. The foremost findings are the significant MR contrast enhancement, unique DNA damaging mode and increased cytotoxicity in the presence of GSH. The former guarantees the applicability of the nanocomposite as an MR contrast agent for real-time imaging, and the latter two reveal a novel mechanism of action for Pt^IV^ prodrugs loaded on nanoparticles. The role of GSH in cisplatin resistance has been studied extensively in both cell lines and cancer tissues, and an increase in the GSH level has been correlated with cisplatin resistance in ovarian, cervical, lung, embryonal and bladder cancer cell lines due to increased inactivation of the drug.^3^ The results of this study indicate that the basic function of intracellular GSH for nano-based Pt^IV^ prodrugs is different from that for Pt^II^ drugs, which has never been perceived so far. The exceptional cleavage mode of DNA and distinct action of GSH may suggest that the cisplatin resistance related to DNA repair and GSH detoxification could be conquered by functionalized Pt^IV^ prodrugs.

## Supplementary Material

Supplementary information
