# Supplementary material for: Glutathione boosting the cytotoxicity of a magnetic platinum(iv) nano-prodrug in tumor cells
Source: Chem Sci. 2016 Jan 20;7(4):2864–9. doi: 10.1039/c5sc04049c (PMC6054038; doi:10.1039/c5sc04049c)
Supplement: Supplementary file 1 [file SC-007-C5SC04049C-s001.pdf]

Electronic Supplementary Material (ESI) for Chemical Science.
This journal is © The Royal Society of Chemistry 2016

Electronic Supplementary Information (ESI)

Glutathione Boosting the Cytotoxicity of Magnetic Platinum(IV) Nano-prodrug in Tumor Cells

Zhenzhu Zhu, Zenghui Wang, Yigang Hao, Chengcheng Zhu, Yang Jiao, Huachao Chen, Yun-Ming Wang, Jun Yan, Zijian Guo* and Xiaoyong Wang*

1. Supplementary figures and tables

Table S1. The cellular uptake of Fe and Pt determined by ICP-MS after HeLa cells (10^6 cells) were incubated with HSPt (20 μM), PEG-SPIONs (20 μg/mL Fe), and HSPt-PEG-SPIONs (20 μM Pt) for 12 h, respectively. Data are expressed as mean (ng) ± standard deviation (S.D.) of at least three independent experiments, with untreated cells as the control.

Metal	HSPt	PEG-SPIONs	HSPt-PEG-SPIONs
Fe (ng)	—	1096.20 ± 10.89	1172.40 ± 12.93
Pt (ng)	614.55 ± 4.73	—	865.05 ± 4.28

Table S2. Loading ratios of Pt to Fe for HSPt-PEG-SPION samples with different initial reaction ratios measured by ICP-MS.

Sample	Reaction molar ratio ($Fe:NH_2:Pt$)	Pt/Fe (w/w)
HSPt-PEG-SPION-1	100:15:100	0.200
HSPt-PEG-SPION-2	100:15:200	0.162
HSPt-PEG-SPION-3	100:15:50	0.052

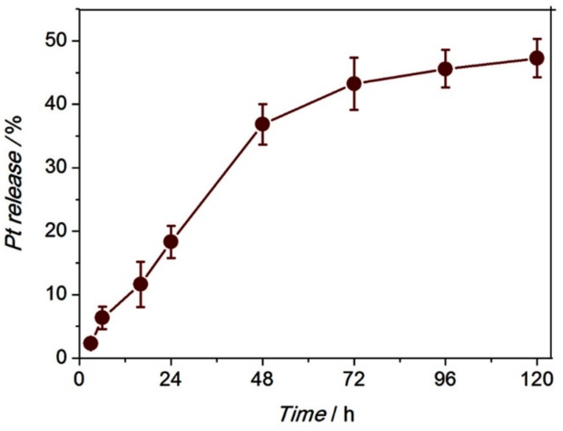

Fig. S1. Time-dependent Pt release from HSPt-PEG-SPIONs (54 μM Pt) determined by ICP-MS after incubation with GSH (2 mM) in PBS buffer (pH 7.4).

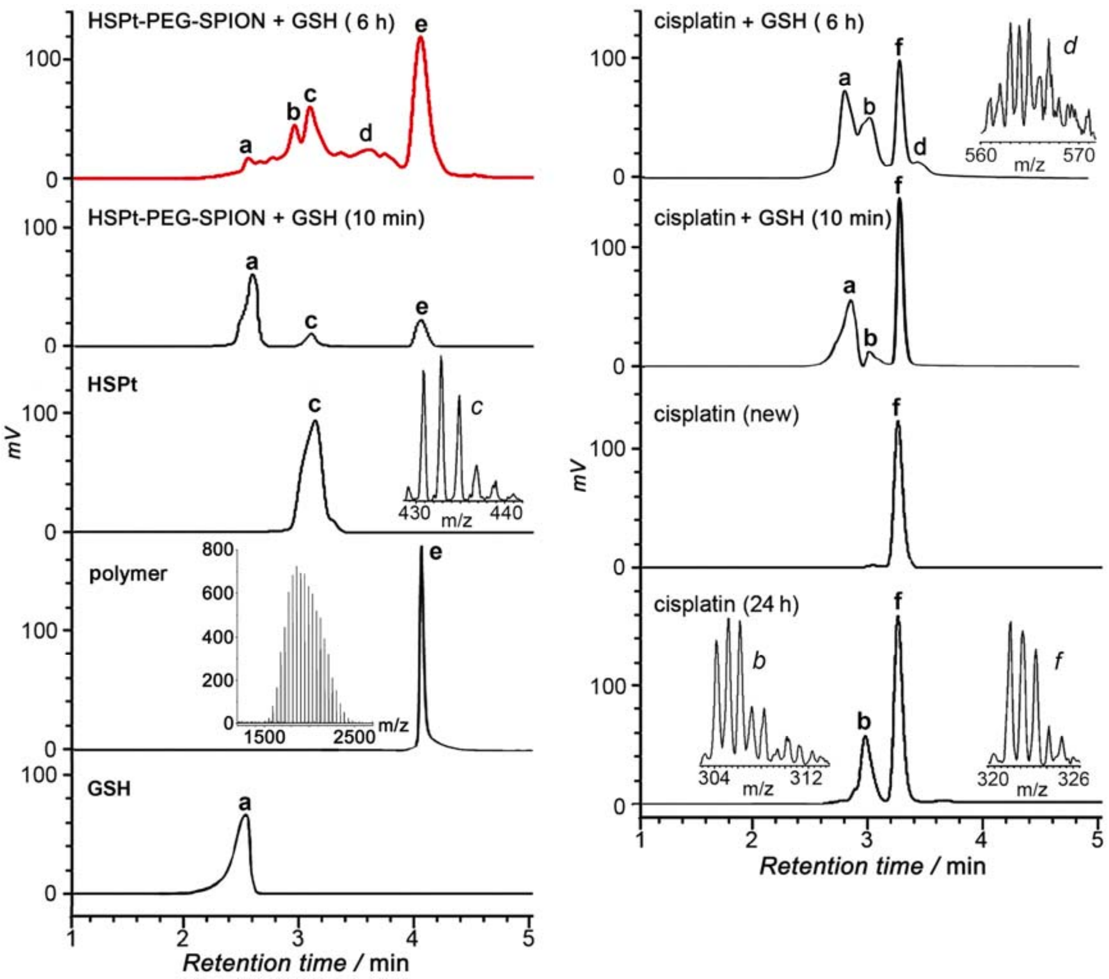

Fig. S2. HPLC spectra for the products of reaction between GSH (2 mM) and HSPt-PEG-SPION or cisplatin (54 μM, in terms of Pt), with those of HSPt, cisplatin, silane-diethyltriamine-methoxy PEG and GSH as references. Insets are enlarged ESI-MS spectra for different platinum complexes or MALDI-TOF-MS spectrum for polymer (see Table S3).

Table S3. Assignments of the major peaks observed in HPLC and ESI-MS spectra (see Fig. S2).

Peak	tR / min	Obsd m/z	Attribution	Formula	Calcd m/z
**a**	2.74	308.17–310.17	$[GSH + H]^+$	$C_{10}H_{18}N_3O_6S$	308.09
**b**	3.03	304.17–312.17	$[Pt(NH_3)_2Cl (OH) + Na]^+$	$H_7N_2OClPtNa$	304.59
**c**	3.18	430.93–437.98	$[Pt(NH_3)_2Cl_2(OH)(O_2CCH_2CH_2CO_2)]^-$	$C_4H_{11}N_2O_5Cl_2Pt$	431.97
**d**	3.44	561.00–571.00	$[Pt(NH_3)_2(GS)H_2O]^+$	$C_{11}H_{25}N_5O_7PtS$	566.49
**e**	4.06	—	silane-diethyltriamine-methoxy PEG	—	—
**f**	3.31	320.17–325.17	$[Pt(NH_3)_2Cl_2 + Na]^+$	$H_6N_2Cl_2PtNa$	321.94

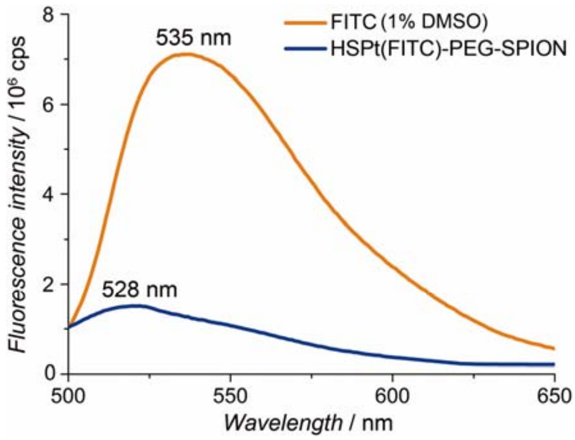

Fig. S3. The fluorescence spectra of FITC and HSPt(FITC)-PEG-SPIONs (λ_{ex} = 477 nm) recorded in water.

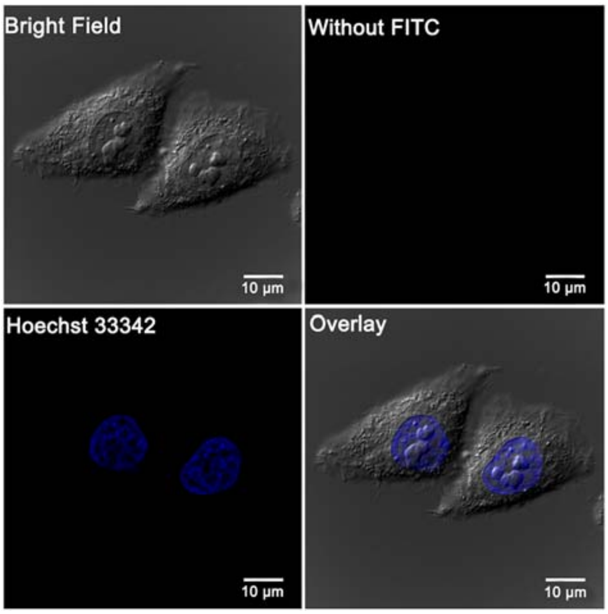

Fig. S4. Photomicrographs of HeLa cells observed by confocal microscopy under FITC excitation.

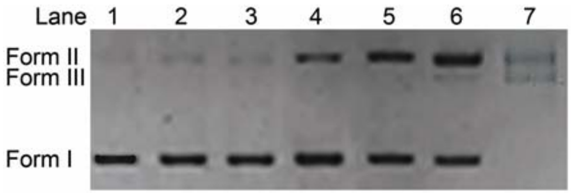

Fig. S5. Agarose gel electrophoresis patterns of supercoiled pUC19 plasmid DNA (20 ng μL^{-1}) after incubation with HSPt-PEG-SPIONs (54 μM in terms of Pt) and different concentrations of GSH at pH 7.4 (50 mM Tris-HCl, 50 mM NaCl) and 37 °C for 16 h. Lane 1, DNA control; lanes 2–7, DNA + HSPt-PEG-SPION + GSH (0, 1, 3, 5, 7, 8 mM, respectively).

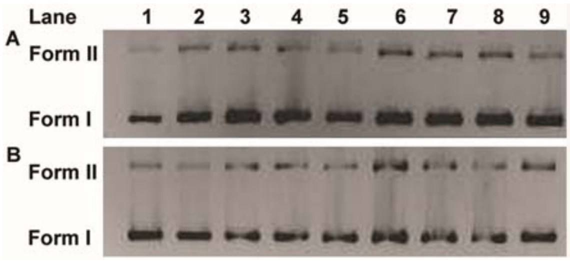

Fig. S6. Agarose gel electrophoretic patterns of supercoiled pUC19 plasmid DNA (20 ng μL^{-1}) after incubation with GSH (2 mM) and different concentrations of A) HSPt or B) cisplatin in buffer (50 mM Tris-HCl, 50 mM NaCl, pH 7.4) at 37 °C for 16 h. Lane 1, DNA control; lanes 2–9, DNA + GSH + HSPt or cisplatin (6, 12, 24, 30, 36, 42, 48, 54 μM, respectively).

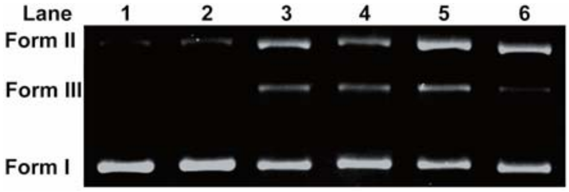

Fig. S7. Agarose gel electrophoresis patterns of supercoiled pBR322 plasmid DNA (20 ng μL^{-1}) after incubation with GSH (10 mM), HSPt-PEG-SPIONs (120 μM) and radical scavenger in buffer (pH 7.4, 50 mM Tris–HCl, 50 mM NaCl) at 37 °C for 16 h. Lane 1, DNA; Lane 2, DNA + GSH; Lane 3, DNA + GSH + HSPt-PEG-SPIONs; Lane 4, DNA + GSH + HSPt-PEG-SPIONs + 10% DMSO; Lane 5, DNA + GSH + HSPt-PEG-SPIONs + 10 mM KI; Lane 6, DNA + GSH + HSPt-PEG-SPIONs + 10 mM NaN_3.

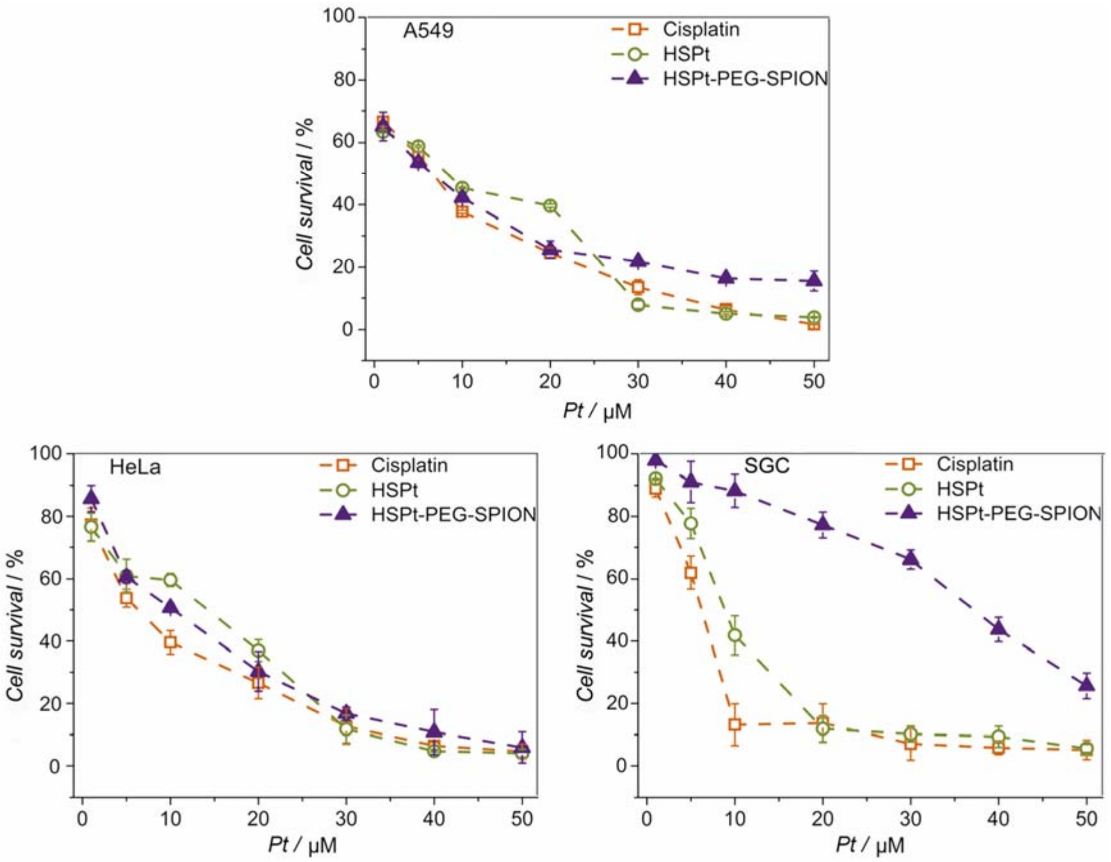

Fig. S8. Cytotoxicity of HSPt-PEG-SPIONs, HSPt, and cisplatin against A549, HeLa, and SGC-7901 cancer cell lines at 48 h. Data are expressed as mean (%) ± standard deviation (S.D.) of at least three independent assays.

Table S4. The IC_{50} values (μM) of HSPt-PEG-SPIONs, HSPt and cisplatin towards different cancer cell lines at 48 h.

Cell line	HSPt-PEG-SPIONs	HSPt	cisplatin
A549	6.55 ± 3.84	8.28 ± 0.62	6.67 ± 1.80
HeLa	10.34 ± 4.56	14.24 ± 0.76	6.40 ± 0.38
SGC-7901	37.52 ± 2.43	8.90 ± 0.57	6.27 ± 0.13

2. Experimental

2.1. Chemical reagents

Iron(III) acetylacetonate, benzyl ether, 1,2-hexadecanediol, oleylamine, oleic acid, methoxy poly(ethylene glycol) (mPEG, Mn = 2000), acryloyl chloride, 3-aminopropyltriethoxysilane (APTES), triethylamine (TEA), trifluoroacetic acid (TFA), fluorescein isothiocyanate (FITC), dichloromethane (DCM), *N*,*N*-dimethylformamide (DMF), tetrahydrofuran (THF), dimethyl sulfoxide (DMSO), hydrogen peroxide (H_2O_2, 30%), reduced glutathione (GSH), ethidium bromide (EB), 1-ethyl-3-(3-dimethylaminopropyl)carbodiimide (EDC), *N*-hydroxysuccinimide (NHS), 3-(4,5-dimethylthiazol-2-yl)-2,5-diphenyltetrazolium bromide (MTT) and buthionine sulfoximine (BSO) were purchased from Sigma-Aldrich and used as received without further purification. Cisplatin was purchased from Shandong Boyuan Pharmaceutical Co., Ltd.

2.2. Cell lines and animals

Human normal liver cell HL-7702, human non-small lung cancer cell A549, human cervical cancer cell HeLa and human gastric cancer cell SGC-7901 were purchased from American Type Culture Collection (ATCC). B6 mice were obtained from the Model Animal Research Center of Nanjing University.

2.3. Instruments

The transmission electron microscopy (TEM) image was obtained using a JEOL JEM-2100 transmission electron microscope at an accelerating voltage of 200 kV. Zeta potential (ζ) was measured in water by using a Malven Nano-Z instrument. Hydrodynamic diameters were determined using a BI-200SM dynamic light scattering system (DLS, Brookhaven Instruments Co., Holtsville, NY). X-ray powder diffraction (XRD) patterns were recorded on a Japan Shimadzu XRD-6000 diffractometer in the 2θ range of 10−90° with Cu K_α radiation (λ = 0.15418 nm) and a scanning rate of 0.05 deg s^{-1}. Field-dependent magnetization was measured on the superconducting quantum interference device (SQUID, Quantum Design, MPMS-XL-7T) magnetometer at 300 K. IR spectra (KBr pellets) in the range of 500–4000 cm^{-1} were recorded on a Bruker VECTOR22 spectrometer. Thermal gravimetric analysis (TGA) was performed on a Pyris TGA instrument with a heating rate of 20 °C min^{-1} in a nitrogen flow (20 mL min^{-1}). X-ray photoelectron spectra (XPS) were acquired on a Thermo ESCALAB 250 electron spectrometer with 150 W monochromatized Al K_α radiation (1486.6 eV), where all peaks were referred to the

signature C1s peak for adventitious carbon at 284.8 eV. Electrospray ionization mass spectra (ESI-MS) were obtained using an LCQ spectrometer (Thermo Scientific). The isotopic distribution patterns were simulated using the ISOPRO 3.0 program. High resolution mass spectra (HR-MS) were measured on a liquid chromatography mass spectrometry instrument-G6500 (Agilent). MALDI-TOF-MS spectrum was obtained on an MALDI-TOF/TOF mass spectrometer (Autoflex II, Bruker). ^{1}H-NMR experiments were performed on a Bruker DRX-500 spectrometer at 298 K. UV-Vis absorption spectra were recorded on a Perkin-Elmer Lambda-35 UV-Vis spectrophotometer using quartz cuvettes (1.0 cm). Fluorescence spectra were recorded on an LS-50B spectrofluorimeter (Perkin-Elmer, USA). The contents of Pt and Fe were determined on an inductively coupled plasma mass spectrometer (ICP-MS) using a standard Plasma-Quad II instrument (VG Elemental, Thermo OptekCorp.). Fluorescence confocal imaging was carried out on a laser scanning confocal imaging system (Olympus TH4-200) consisting of ZEISS Laser Scanning Microscope (LSM 710) and a 20 mW-output 488 nm argon ion laser. Transverse relaxation time (T_2) was measured at 0.52 T, 21.96 MHz, and 32 °C on NMI20 Analyst (Shanghai Niumag Corporation, China) equipped with a coil (inner diameter, 15 mm) using an inversion recovery pulse sequence. T_2-weighted MRI was taken on a Siemens Tim 3.0 T MRI scanner (East China Normal University, Shanghai) equipped with a home-made small animal radio frequency coil (inner diameter, 50 mm). Image reconstruction and analysis were performed using Syngo fastView software (Siemens, Munich, Germany) and Image J (version 1.43, NIH).

2.4. Synthesis of SPIONs (Fe_3O_4)

SPIONs were synthesized referring to the reported method.[1] Specifically, iron(III) acetylacetonate (2.1191 g, 6 mmol) in benzyl ether (5 mL) was mixed with 1,2-hexadecanediol (2.5844 g, 10 mmol), oleylamine (2 mL, 6 mmol) and oleic acid (1.92 mL, 6 mmol). The mixture was heated to 120 °C for 10 min, 200 °C for 2 h, and 300 °C for 1.5 h under N_2, sequentially. The reaction mixture was cooled to room temperature and the black product was precipitated by acetone (20 mL). The product was separated by centrifugation and a magnet, dispersed in chloroform (10 mL) and stored at 4 °C for later use.

2.5. Synthesis of PEG-SPIONs

Silane-diethyltriamine-methoxy poly(ethylene glycol) (silane-DETA-mPEG) was synthesized as reported previously.[2] The silane-DETA-mPEG stabilized SPIONs were obtained by ligand exchange reaction. Specifically, SPIONs (23 mg) were dispersed in toluene (20 mL) and mixed with silane-DETA-mPEG (230 mg). The resulting solution was sonicated for 6 h and the silane-DETA-mPEG coated NPs were precipitated by hexane. After decanting the supernatant, the precipitate was re-dispersed in hexane for three times. The particles were then dispersed in water and purified by dialysis against water (MWCO 8000Da). To remove the excess reagents and byproducts, the NPs were subjected to magnetic separation. The obtained PEG-SPIONs were then concentrated and reconstituted in water, and stored at 4 °C for further use. The primary amino groups available on the surface of NPs were determined by the ninhydrin assay.[3] After reaction, the absorbance of the solution was measured on a UV-Vis spectrometer at 570 nm. The concentration of amino groups was obtained from the calibration curve, using lysine as a standard. The content of Fe in PEG-SPIONs was determined by ICP-MS.

2.6. Synthesis of c,t,c-[$Pt(NH_3)_2Cl_2(OH)(O_2CCH_2CH_2CO_2H)$] (HSPt)

Hydrogen peroxide (30%, 20 mL) was added dropwise to the aqueous suspension of cisplatin (0.4 g, 1.33 mmol, 12 mL) at 60 °C. After reaction for 4 h, the bright yellow solution was cooled at room temperature overnight to give yellow crystals of *c,c,t*-$[Pt(NH_3)_2Cl_2(OH)_2]$. The crystals were filtrated and washed with icy water. Yield: 65.6% (0.291 g, 0.872 mmol). IR (KBr, cm^{-1}): 3460 (s, OH stretch), 1076 (m, Pt-OH bend), 557 (m, Pt-N(O) stretch). ^{195}Pt-NMR (500 MHz, D_2O, δ, ppm): 892.61. ESI-MS (positive mode, m/z): $[Pt(NH_3)_2Cl_2(OH)_2 + H]^+$, 335.42, calcd 335.06; $[2Pt(NH_3)_2Cl_2(OH)_2 + H]^+$, 669.00, calcd 669.12. The suspension of *c,c,t*-$[Pt(NH_3)_2Cl_2(OH)_2]$ (0.2 g, 0.6 mmol) in DMSO (16 mL) was added to succinic anhydride (0.06 g, 0.6 mmol) and the mixture was stirred overnight to afford a bright yellow solution. The solution was concentrated to ~ 0.5 mL in vacuum and added to 10 mL icy acetone. The resulting pale yellow precipitate was filtrated, washed with acetone and dried in vacuum. Yield: 97.2% (0.253 g, 0.583 mmol). ^{1}H-NMR (400 MHz, DMSO-d^6, δ, ppm): 5.94 (m, 6H, NH_3); 2.39 (m, 4H, CH_2). ^{195}Pt-NMR (500 MHz, D_2O, δ, ppm): 1043.30. HR-MS (negative mode, m/z): $[Pt(NH_3)_2Cl_2(OH)(O_2CCH_2CH_2CO_2)]^-$, 430.9817–437.9817, calcd 431.9693; $[Pt(NH_3)_2Cl_2(OH)(O_2CCH_2CH_2CO_2H) + Na]^+$, 454.9639–460.9639, calcd 455.9669.

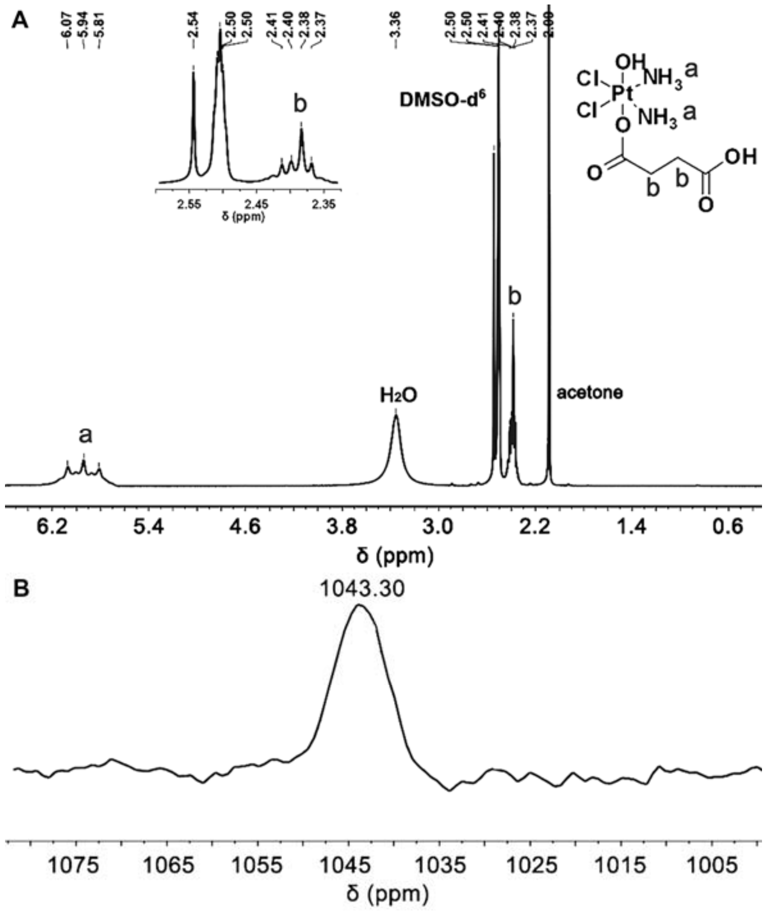

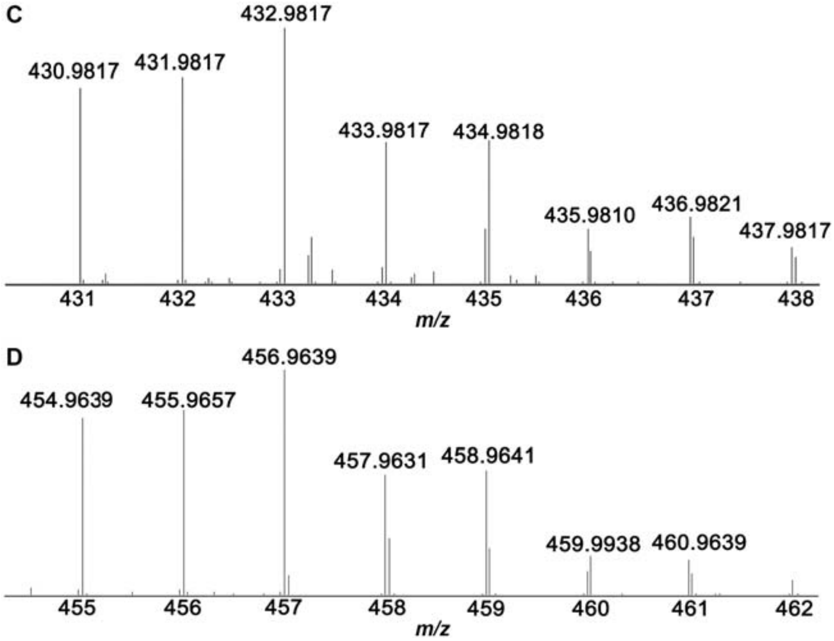

Fig. S9. A) ^{1}H-NMR (400 MHz, DMSO-d^{6}) and B) ^{195}Pt-NMR (500 MHz, D_2O) spectra of HSPt; C) HR-MS spectra of $[Pt(NH_3)_2Cl_2(OH)(O_2CCH_2CH_2CO_2)]^{-}$ and D) $[Pt(NH_3)_2Cl_2(OH)(O_2CCH_2CH_2CO_2H) + Na]^{+}$.

2.7. Synthesis of HSPt-PEG-SPIONs

HSPt (43.3 mg, 0.1 mmol), EDC (0.38 mg, 0.1 mmol), and NHS (0.38 mg, 0.1 mmol) were dissolved in DMSO (2 mL) and allowed to react for 30 min at room temperature. PEG-SPIONs (13 mg, 10 μmol in terms of NH_2) was dispersed in water (5 mL) and added to the reaction solution. The mixture was sonicated for 6 h in the absence of light. Amide linkages were readily formed in the presence of EDC and NHS, resulting in HSPt-PEG-SPIONs. Excess EDC was removed by dialysis against water (MWCO 12~14 kDa). After purification, HSPt-PEG-SPIONs were dissolved in water for later use.

2.8. Synthesis of HSPt(FITC)-PEG-SPIONs

HSPt-PEG-SPIONs with surplus amino groups (10 μmol Fe, 1.46 μmol NH_2, 0.58 μmol Pt) were dissolved in coating buffer (0.05 M sodium carbonate and 0.1 M sodium bicarbonate). FITC (10 μL, 10 mg mL^{-1} in DMSO, 0.26 μmol) was added to the suspension and stirred at 4 °C overnight. Free FITC and DMSO were removed by dialysis (MWCO 30 kDa) against deionized water. HSPt(FITC)-PEG-SPIONs were purified by magnetic separation and stored at 4 °C in the dark.

Scheme S1. Synthesis route to HSPt-PEG-SPION and HSPt(FITC)-PEG-SPION.

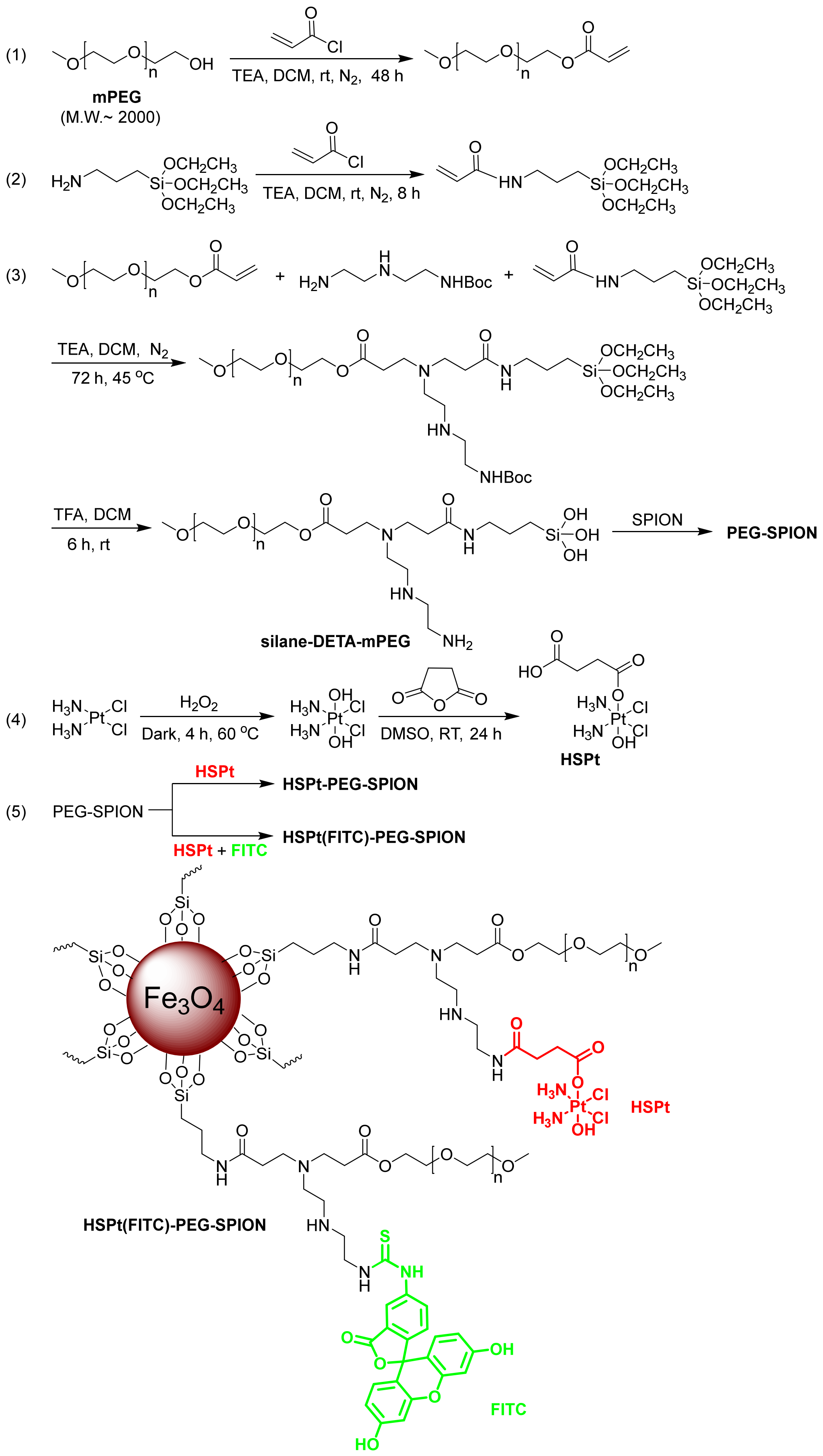
(1)
mPEG
(M.W.~ 2000)
TEA, DCM, rt, N2, 48 h
(2)
TEA, DCM, rt, N2, 8 h
(3)
NHBoc
TEA, DCM, N2
72 h, 45 °C
TFA, DCM
6 h, rt
SPION
PEG-SPION
silane-DETA-mPEG
(4)
H2O2
Dark, 4 h, 60 °C
DMSO, RT, 24 h
HSPt
(5)
PEG-SPION
HSPt
HSPt-PEG-SPION
HSPt(FITC)-PEG-SPION
HSPt + FITC
Fe3O4
HSPt
HSPt(FITC)-PEG-SPION
FITC

2.9. Transverse relaxation (r_2) and in vitro MRI

The transverse relaxation time (T_2) and the MR images of HSPt-PEG-SPION aqueous suspensions (0.11, 0.22, 0.44, 1.08, and 1.61 mM in terms of Fe) were measured at 0.52 T, 21.96 MHz, and 32 °C on NMI20 Analyst. Experimental settings are as follows: P90 = 5.00 μs, P180 = 11.00 μs, SW = 100 KHz, D3 = 0.08 μs, TR = 1000 ms, RG1 = 20, RG2 = 3, NS = 2, TE = 0.4 ms, NECH = 1000; FOV Read = 100 mm, FOV Phase = 100 mm, Slices = 1, Slice width = 4 mm, K space = 192 × 256; TR = 1500 ms, TE = 8.5 ms. The transverse relaxivity (r_2) was calculated according to the following equation: $r_2[\mathrm{Fe}] = 1/T_2 - 1/T_2^0$, where $1/T_2$ is the relaxation rate in the presence of HSPt-PEG-SPIONs, $1/T_2^0$ is the relaxation rate of pure water, and [Fe] is the concentration of HSPt-PEG-SPIONs in terms of Fe.

The samples for *in vitro* MRI were obtained by incubating HeLa cells (1×10^6) with HSPt-PEG-SPIONs (0, 10, 50, 100 μg mL^{-1}, in terms of Fe) at 37 °C for 18 h, respectively. The cells were collected and dispersed in 1% argose, and T_2-MR images were acquired at 0.52 T, 21.96 MHz, and 32 °C on NMI20 Analyst.

2.10. In vivo MRI

B6 mice (female, 9 weeks, *ca.* 20 g, n = 5) were used under the protocols approved by the Model Animal Research Center of Nanjing University. RM1 murine prostate cancer cells (1×10^6 cells) were suspended in PBS (100 μL) and subcutaneously inoculated into the shoulder of each mouse. About 2 weeks later, the tumor-bearing (~ 276 mm^3) mice were randomized into 2 groups, anesthetized with 1–2% inhaled isoflurane anesthesia and intravenously injected with HSPt-PEG-SPIONs (200 μL, 3 mg Fe/kg body weight) for MRI. A small magnet was attached to the tumor area after the injection, using a mouse without magnet as the control. After 2 or 3 h, the magnet was removed and the animal was subjected to T_2-weighted MRI on a 3.0 T clinical MRI instrument (Simens, Magnetom Trio Tim 3.0 T) equipped with a special coil designed for small animals. The tumor size was measured by a caliper and calculated approximately (tumor volume = length × width^2/2). A spin-echo sequence was used with the following parameters: echo time TE = 34 ms, slice thickness SL = 1.2 mm, repetition time TR = 6000 ms, FOV = 47 × 47 mm^2. Slice orientation was coronal. Image reconstruction and analysis were performed and the signal intensity of the MR images was ascertained by the average intensity in the defined region of interest. The contrast enhancement (%) was calculated by the following equation:

$$\text{Enhancement (\%)} = (\mathrm{SI} - \mathrm{SI}_0) / \mathrm{SI}_0 \times 100$$

where SI is the signal intensity of the tumor area after the treatment with HSPt-PEG-SPIONs, and SI_0 is the signal intensity of the tumor area at the beginning of the treatment. After MRI, the mice were sacrificed while they were under anesthesia.

2.11. Drug release

HSPt-PEG-SPIONs (54 μM Pt) were incubated with GSH (2 mM) in PBS buffer (pH 7.4, 40 mL) in a dialysis bag (MWCO 3000, Fisher) and were dialyzed under constant shaking. Aliquot sample solution (2 mL) was collected from the external aqueous milieu of the device at regular time interval and the Pt content was measured by ICP-MS. After sampling, equal volume (2 mL) of fresh PBS buffer was immediately added into the incubation medium. The released drug content was expressed as the percentage of cumulative Pt outside the dialysis bag to the total Pt in

the NPs.

Otherwise, HSPt-PEG-SPIONs (54 μM Pt, Pt/Fe = 20 %, wt) were incubated with GSH (2 mM) at 37 °C for 6 h. The sample was centrifuged and the supernatant was subjected to analysis at 230 nm via a Beckman Coulter HPLC instrument equipped with a C18 reverse phase column (eluent: H_2O/CH_3CN, 95/5). ESI-MS was used to characterize the peaks appeared in the HPLC spectra. Cisplatin (2 mM) was incubated with GSH (2 mM) in 37 °C for 6 h as a control.

2.12. Cellular uptake

HeLa cells were seeded at a density of 2×10^5 cells per well on cover glasses and cultured in 5% CO_2 at 37 °C for 24 h. The cells were then incubated with HSPt(FITC)-PEG-SPION (20 μM Pt) for 12 h at 37 °C, washed three times in PBS, and fixed with 4% formaldehyde solution for 30 min at room temperature. The cell nuclei were stained with Hoechst 33324. The cover glasses containing fixed cells in a mixture of PBS and glycerol (1:1) were mounted on a microscope slide. The cells were observed by confocal fluorescence microscopy.

2.13. Interaction with DNA

Supercoiled pUC19 DNA (20 ng μL^{-1}) was treated with gradient concentrations of HSPt-PEG-SPIONs in the presence of GSH in Tris-HCl buffer (50 mM Tris-HCl/50 mM NaCl, pH 7.40). The mixtures (10 μL) were incubated at 37 °C for 16 h and the reactions were quenched by 2 μL of loading buffer (30 mM EDTA, 36% glycerol, 0.05% xylene cyanol FF, and 0.05% bromophenol blue). The resulting solutions were loaded onto the native agarose gel (1%) and subjected to electrophoresis in a TAE buffer (40 mM Tris acetate, 1 mM EDTA). The resultant gel was stained with ethidium bromide (0.5 mg mL^{-1}) in the TAE buffer and visualized using an UVP gel doc system.

Radical scavengers such as DMSO (1 μL, 10%), KI (1 μL, 10 mM) and NaN_3 (1 μL, 10 mM) were added to the buffer solutions (50 mM Tris-HCl/50 mM NaCl, pH 7.4) containing supercoiled pBR322 DNA (20 ng μL^{-1}) prior to the addition of HSPt-PEG-SPIONs (3 μL, 120 μM) with or without GSH (1 μL, 10 mM), respectively. The mixture was incubated at 37 °C for 16 h. The quenching and electrophoresis were performed as described above. The quantification of each DNA form was accomplished by Image J analysis.

2.14. Cytotoxicity assay

Human normal liver cell line HL-7702, human cancer cell lines A549, HeLa, and SGC-7901 were cultured overnight after inoculation in RPMI-1640 medium supplement. All the cultures were maintained in an incubator in a highly humidified atmosphere of 95% air with 5% CO_2 at 37 °C. The cytotoxicity of HSPt-PEG-SPION, HSPt, and cisplatin was tested by the MTT assay as we described recently elsewhere.[4]

2.15. Analysis of cellular GSH

The concentration of intracellular GSH was measured by a GSH Assay Kit (Beyotime, China). Briefly, cell extracts were prepared according to the manufacturer's instructions. GSH was detected by measuring the product of 5-thio-2-nitrobenzoic acid by colorimetric analysis at 412 nm. The cellular GSH contents were calculated using the standard curve generated in parallel

experiments. After the cells were treated with HSPt-PEG-SPIONs for 48 h, GSH was retested in the same way. To confirm the promotive effect of GSH to the cytotoxicity, A549 cancer cells (10^6) were seeded in the 96 wells plate and treated with 200 μM BSO for 24 h. The cells were then treated with HSPt-PEG-SPIONs for 48 h and the survival ability was examined by the MTT assay.

References

1. S. H. Sun and H. Zeng, *J. Am. Chem. Soc.*, 2002, **124**, 8204–8205.
2. S. -C. Wu, K. -L. Lin, T. -P. Wang, S. -C. Tzou, G. Singh, M. -H. Chen, T. -L. Cheng, C. -Y. Chen, G. -C. Liu, T. -W. Lee, S. -H. Hu and Y. -M. Wang, *Biomaterials*, 2013, **34**, 4118–4127.
3. N. C. Dubey, B. P. Tripathi, M. Stamm and L. Ionov, *Biomacromolecules*, 2014, **15**, 2776–2783.
4. Z. Z. Zhu, X. Y. Wang, T. J. Li, S. Aime, P. J. Sadler and Z. J. Guo, *Angew. Chem. Int. Ed.*, 2014, **53**, 13225–13228.
